# Modulating the expression of tumor suppressor genes using activating oligonucleotide technologies as a therapeutic approach in cancer

**DOI:** 10.1016/j.omtn.2022.12.016

**Published:** 2022-12-27

**Authors:** Georgina L. Gregory, Ian M. Copple

**Affiliations:** 1 Department of Pharmacology & Therapeutics, Institute of Systems, Molecular & Integrative Biology, University of Liverpool, Liverpool L69 3GE, UK

**Keywords:** MT: oligonucleotides: therapies and applications, oligonucleotide, cancer, tumor suppressor gene, saRNA, mRNA

## Abstract

Tumor suppressor genes (TSGs) are frequently downregulated in cancer, leading to dysregulation of the pathways that they control. The continuum model of tumor suppression suggests that even subtle changes in TSG expression, for example, driven by epigenetic modifications or copy number alterations, can lead to a loss of gene function and a phenotypic effect. This approach to exploring tumor suppression provides opportunities for alternative therapies that may be able to restore TSG expression toward normal levels, such as oligonucleotide therapies. Oligonucleotide therapies involve the administration of exogenous nucleic acids to modulate the expression of specific endogenous genes. This review focuses on two types of activating oligonucleotide therapies, small-activating RNAs and synthetic mRNAs, as novel methods to increase the expression of TSGs in cancer.

## Graphical abstract

**Figure:**
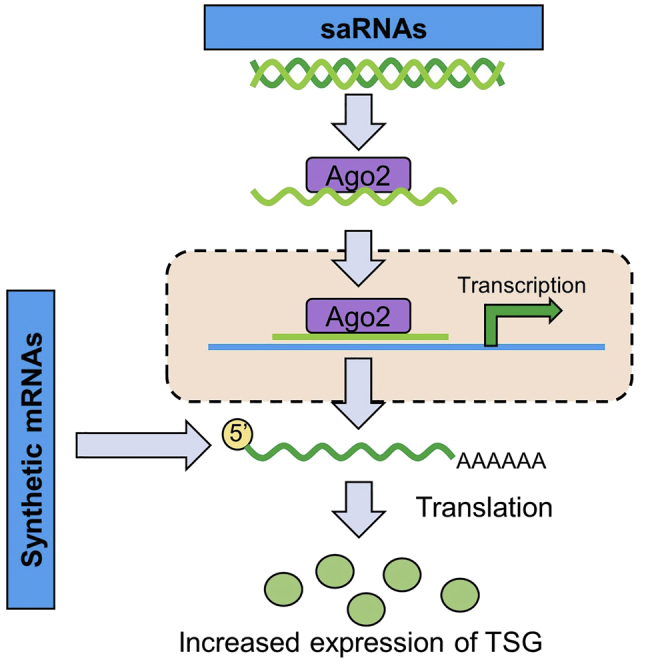

## Teaser

TSGs are genes that are commonly downregulated in cancer. An emerging classification of drugs, called oligonucleotide therapies, have the ability to upregulate or restore the expression of TSGs. This review summarizes saRNAs and synthetic mRNAs and their capability to modulate TSG expression.

## Introduction

### What are tumor suppressor genes?

Tumor suppressor genes (TSGs) are a category of genes that serve to keep cell growth tightly regulated, such that a cell will only divide when absolutely necessary and in response to the appropriate external signals, such as growth factors. In addition to controlling proliferation, TSGs are also involved in preventing cells from migrating to, and invading, other tissues, as well as stimulating cells to undergo apoptosis when they encounter a cellular stress, such as DNA damage. If the latter is left unchecked, this could result in the introduction of mutations and dysregulation of the cell cycle. When TSG function is lost, this can result in critical cellular processes becoming dysregulated and cells may proliferate uncontrollably, fail to initiate apoptosis in response to damage, or start to invade through the basement membrane and metastasize to a different part of the body.^1^ TSGs are, therefore, an important group of genes in the context of cancer pathogenesis and therapy.

Although TSGs are a vast group of genes, they can be further categorized according to their function and the pathways which they control, see below and Table 1. TSGs have traditionally been labeled as being homozygous recessive in terms of their role in promoting carcinogenesis, meaning that both alleles of the TSG have to exhibit loss of function to result in loss of protein activity and promotion of cell proliferation and tumorigenesis. The “two-hit hypothesis” was first defined during the analysis of retinoblastoma (RB), a cancer of the eye in children.^38^ Knudson^38^ reported that familial RB was dominantly inherited. Indeed, it was shown that children inherit a mutation in the *RB1* gene, which encodes the RB protein (although the actual disease-causing gene was not identified at the time), from one parent, which predisposes them to developing RB. A secondary mutation in the other *RB1* allele is acquired somatically as the eye develops, meaning that both alleles of the gene are mutated, leading to a loss of function. This means that there is no functional RB protein in the retinoblast cell; therefore, it cannot exert its function as a repressor of cell cycle progression. The deficient cells are, therefore, able to progress through the cell cycle, even in the absence of the appropriate growth factor signal. This results in the initiation of tumorigenesis.

**Table 1:** Summary of studies investigating upregulation of TSGs using saRNAs in cancers

Classification	Gene targeted for upregulation using saRNAs	Protein encoded by gene	Function of tumor suppressor	Cancer(s) in which upregulation has been investigated	Stage of development	Reference(s)
Cell-cycle arrest	*CDKN1A*	P21	Activated by p53 in response to DNA damage, *CDKN1A* is a cyclin-dependent kinase inhibitor which prevents arrests cells in G1/S-phase of the cell cycle.	Colorectal cancer, HCC, Prostate cancer, Bladder cancer	*In vivo* (Colorectal) *In vivo* (Prostate) *In vitro* (HCC) *In vitro* (Bladder)	Li et al., Wu et al., Kang et al., Place et al., Wang et al., Kosaka et al., Zhang et al., Wu et al., Chen et al. (Refs.^2^^,^^3^^,^^4^^,^^5^^,^^6^^,^^7^^,^^8^^,^^9^^,^^10^)
	*TP53*	P53	Commonly referred to as the guardian of the genome, p53 is activated in response to cellular stress to induce cell-cycle arrest, apoptosis, DNA repair and cell senescence.	Bladder cancer, Pheochromocytoma	*In vivo* (Bladder)*In vivo* (Pheochromocytoma)	Wang et al., Lin et al. (Refs.^11^^,^^12^)
	*VHL*	Von Hippel-Lindau protein	Forms part of an E3 ubiquitin ligase complex, involved in regulating angiogenesis, cell cycle progression and apoptosis.	Renal cell carcinoma	*In vitro*	Kang et al. (Ref.^13^)
Inhibit pro-proliferative signaling pathways	*CEBPA*	CCAAT/enhancer-binding protein alpha	Transcription factor which prevents over-proliferation of mature hepatocytes and helps maintain healthy hepatocyte function.	HCC PDAC	Phase II clinical trials (HCC) *In vivo* (PDAC)	Huan et al., Sarker et al., Hashimoto et al., Yoon et al., Reebye et al.(Refs.^14^^,^^15^^,^^16^^,^^17^^,^^18^^,^^19^)
	*INTS6*	Integrator complex subunit 6	Able to interact with RNA polymerase II and is involved in the processing of small nuclear RNAs. Can downregulate the pro-proliferative Wnt signaling pathway.	Prostate cancer	*In vitro*	Chen et al. (Ref.^20^)
	*HIC1*	Hypermethylated in cancer 1	Transcriptional repressor of pro-proliferative genes such as *CCND1*.	Gastric cancer	*In vitro*	Pan et al. (Ref.^21^)
	*PTEN*	Phosphatase and tensin homologue on chromosome 10	Dephosphorylates PIP_3_ to PIP_2_, preventing activation of AKT/PKB and its downstream effects including proliferation and cell survival.	Lung cancer	*In vitro*	Li et al. (Ref.^22^)
Maintain cell morphology and motility	*CDH1*	E-cadherin	Forms adherens junctions between adjacent cells, preventing epithelial-to-mesenchymal transition. Sequesters β-catenin to the membrane, blocking the pro-proliferative Wnt signaling pathway.	Bladder cancer, Prostate cancer, Renal cell carcinoma, Breast cancer	*In vitro* (bladder) *In vitro* (prostate) *In vitro* (renal) *In vivo* (breast)	Li et al., Dai et al., Wu et al., Li et al., Junxia et al., Mao et al. (Refs.^2^^,^^23^^,^^24^^,^^25^^,^^26^^,^^27^)
	*VEZT*	Vezatin, adherens junctions transmembrane protein	Transmembrane protein involved in maintaining adherens’ junctions.	Gastric cancer	*In vitro*	Xie et al. (Ref.^28^)
	*DPYSL3*	Dihydropyrimidinase like 3	Involved in semaphorin/collapsin-induced signaling, which regulate cell morphology and motility. Prevents cancer cell metastasis.	Prostate cancer	*In vivo*	Li et al. (Ref.^29^)
	*NKX3-1*	NK3 Homeobox 1	Thought to be a prostate-specific tumor suppressor, involved in maintaining normal cellular phenotype.	Prostate cancer	*In vivo*	Ren et al. (Ref.^30^)
Promote apoptosis	*PAWR*	Pro-apoptotic WT1 regulator	Stimulates apoptosis by activating the Fas-mediated apoptotic pathway and inhibiting pro-survival pathways.	Prostate cancer	*In vitro*	Yang et al. (Ref.^31^)
	*FHIT*	Fragile histidine triad	Plays a role in promoting apoptosis by preventing the degradation of p53.	Endometrial cancer	*In vitro*	Zhu et al. (Ref. ^32^)
Function unknown/unclear	*DIRAS1*	Di-Ras1	Member of the guanosine triphosphatase (GTPase) Ras superfamily, specific function is unclear.	Renal cell carcinoma	*In vivo*	Xu et al. (Ref.^33^)
	*KLF4*	Kruppel like factor 4	A zinc-finger transcription factor. Can act as a tumor suppressor or oncogene dependent on the genetic context of the tissue in which it is expressed.	Colorectal cancer, Prostate cancer	*In vitro*	Zhou et al., Wang et al. (Refs.^34^^,^^35^)
	*WT1*	Wilms’ tumor protein	Can act as a tumor suppressor or oncogene, depending on the cell type in which it is expressed, and level of expression.	HCC	*In vitro*	Qin et al. (Ref.^36^)
	*MAS1*	Mas receptor 1	Modulates the renin-angiotensin system to control the tumor microenvironment and inhibit cell migration.	Ovarian cancer, Breast cancer, Pancreatic cancer	*In vivo* (ovarian) *In vivo* (breast) *In vitro* (pancreatic)	Xiong et al. (Ref.^37^)

CCND1, cyclin D1; CDH1, cadherin-1; CDKN1A, cyclin dependent kinase inhibitor 1A; HCC, hepatocellular carcinoma; PDAC, Pancreatic ductal adenocarcinoma; PIP_2,_ phosphatidylinositol 4,5-bisphosphate; PIP_3_, phosphatidylinositol 3,4,5-trisphosphate.

However, many sporadic cancers (i.e., cancers where there are no inherited pre-disposing gene mutations) exhibit a loss of function in a single allele of a TSG, whereas the other allele seems to be normal. These instances led to the development of the concept of haploinsufficiency, which suggests that the loss of a single allele may be sufficient for a TSG to have decreased function and, hence, play a role in the development of a tumor.^39^

The continuum model of tumor suppression takes this concept a step further, and suggests that even subtle changes in the expression of a TSG can impact its function and tumor-suppressive activity.^39^ This model takes into consideration the fact that genes can be regulated in ways other than by mutation or allele loss, such as by epigenetic modifications, microRNAs and post-translational modifications. These changes can result in altered expression of the gene, but the gene itself is not mutated. This means that the transcription and translation of the gene produces a normal, functional protein, but the levels of the protein are lower in the tumor compared with normal, healthy tissue. The continuum model of tumor suppression, therefore, implies that even a slight upregulation in a TSG may recover some of its tumor-suppressive function, and hence stunt the growth, invasiveness, or malignancy of a tumor, depending on the molecular pathways that the TSG acts on.

An example of a TSG that does not follow Knudson’s two-hit hypothesis of tumor suppression is phosphatase and tensin homolog on chromosome 10 (*PTEN*). Studies have shown that subtle changes in the expression level of *PTEN* can result in the loss of its function and an increase in carcinogenesis in certain tissues. For example, a 20% decrease in the normal level of *PTEN* expression is sufficient to cause cancer in the breast,^39^^,^^40^ but is not sufficiently low enough to cause carcinogenesis in the liver, small intestine, pancreas, adrenal glands, and prostate.^40^ Furthermore, haploinsufficiency of *PTEN* is sufficient to accelerate prostate tumor progression in mice.^41^

### TSGs in the clinic

It is now well recognized that cancer is a genetic disease, with the two major categories of genes involved in the initiation of cancer being oncogenes and TSGs. There are many drugs that have been developed that target overactive oncogenes, such as kinase inhibitors like sorafenib in hepatocellular carcinoma, and imatinib in chronic myeloid leukemia.^42^^,^^43^ However, small molecule drugs targeting underactive or mutated TSGs have been somewhat lacking.

There are some examples where this has been the case, such as the identification of thioemicarbazone family molecules in targeting mutant p53, allowing for zinc chelation and the restoration of the DNA-binding properties of the important transcription factor.^44^ Furthermore, compounds such as PhiKan083 allow for the restoration of normal function in p53 mutant cells harboring a Y220C or Y220S mutation by stabilizing the protein structure and preventing denaturation.^45^^,^^46^ However, no drugs directly targeting p53 have yet to make it through clinical trials to approval, indicating the difficulty in targeting TSGs, even after years of extensive research into the gene and protein’s structure and function.

A p53 activator that has entered clinical trials is APR-246, a quinuclidinone derivative that is able to rescue the ability of mutant p53 to interact with DNA by interacting with the cysteine residues within the protein to restore the wild-type conformation.^47^ APR-246 has completed phase I and II trials for multiple different cancer types and in combination with other anti-cancer therapies.^48^^,^^49^^,^^50^ The first-in-human trial showed that the drug does have some promising anti-tumour activity, indicated by increased apoptosis of circulating malignant cells in selected patients, regardless of *TP53* mutation status.^48^ However, the active form of the drug, methylene quinuclidinone, to which APR-246 is converted once inside the body, is rapidly degraded under physiological conditions, which limits the effectiveness of APR-246 as a monotherapy.^51^

Another approach that has been used to circumvent underactive TSGs is the targeting of the downstream consequences of such downregulation. For example, the downregulation or loss of PTEN leads to overactivity of the AKT pathway, which provides an ideal target for small molecule drugs via inhibition of the kinase AKT.^52^ However, oligonucleotide therapies offer an advantage as they allow for the root of the issue (i.e., the underactive TSG) to be targeted directly.

**Figure 1:**
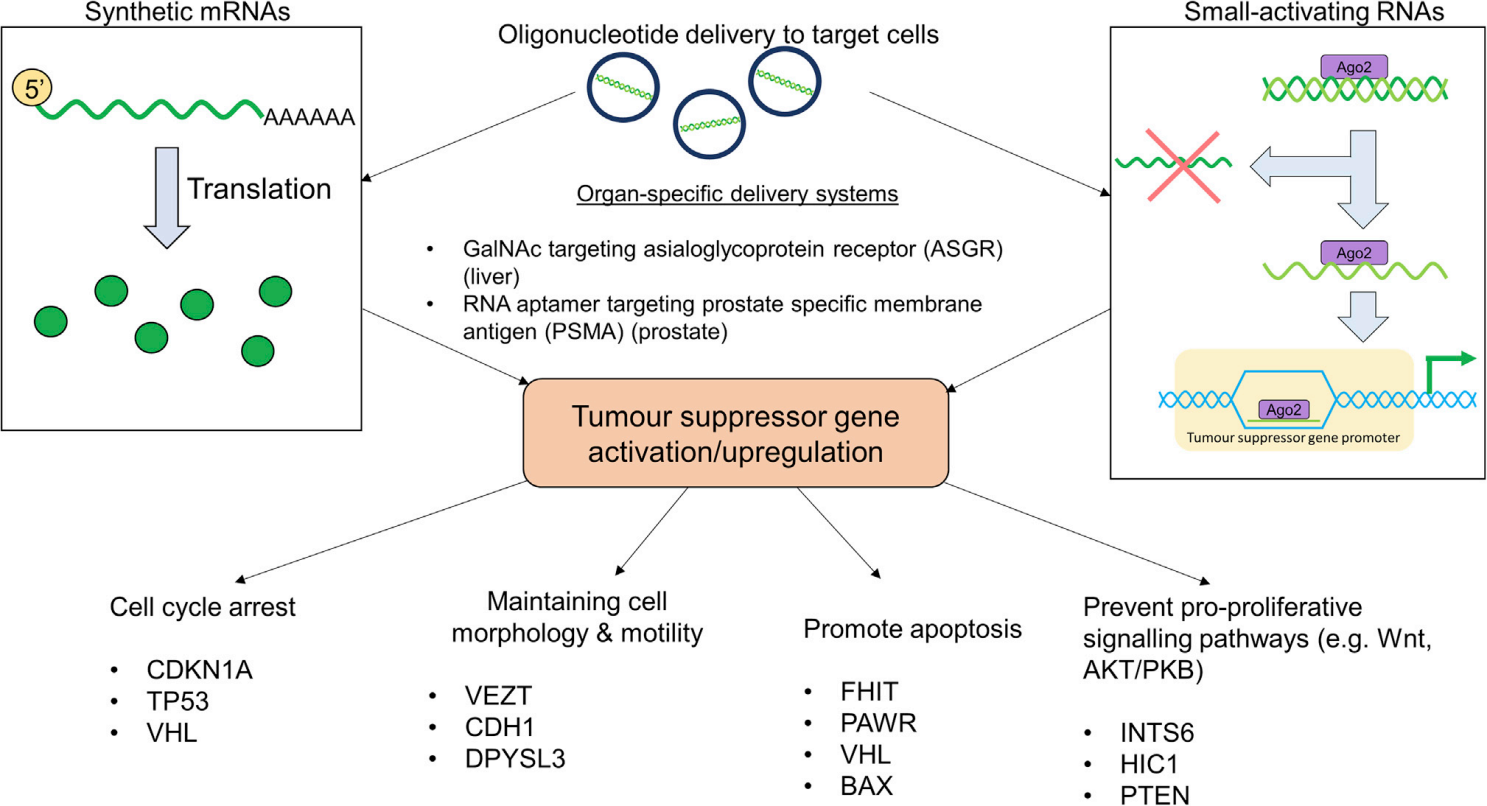
An overview of the use of activating oligonucleotides to target TSGs in cancer Synthetic mRNAs and saRNAs are two types of activating oligonucleotide which have the potential to upregulate TSGs and restore their function. There are challenges with the delivery of such oligonucleotides *in vivo*, and their mechanisms of action differ once inside their target cell. GalNAc, N-acetylgalactosamine.

In light of the critical role of TSG modulation in various forms of cancer, there is much interest in the potential to target specific TSGs as a novel therapeutic approach in patients. This review explores the potential of using oligonucleotide therapies to restore the function of TSGs in cancer (Figure 1), and the benefits and challenges of such an approach.

### What are oligonucleotide therapies?

Oligonucleotide therapies include inhibitory antisense oligonucleotides (ASOs) and short interfering RNAs (siRNAs), along with stimulatory small-activating RNAs (saRNAs) and synthetic, nucleoside-modified mRNAs. Another example of an activating oligonucleotide is plasmid DNA, which is an approach used in gene therapy. Plasmid DNA can be delivered to a host cell via both viral and non-viral methods, with viral-based delivery systems accounting for the majority of gene therapy approaches in clinical trials.^53^ However, such methods of delivery are associated with immunogenicity and the risk of insertional mutagenesis^54^^,^^55^ and are not discussed further in the current review. Broadly, oligonucleotide therapies involve the administration of exogenous nucleic acids to a cell to modulate expression of specific target genes. Oligonucleotides can interact with complementary sequences on RNA or DNA, depending on their mechanism of action, and they offer an advantage over small molecule drugs, which tend to work on protein targets and can lack specificity. Oligonucleotide therapies offer a more specific approach to modulating gene expression, which can minimize off-target effects and toxicity. Given that the exact sequence of the oligonucleotide is known, and the fact that these therapies interact with their target via complementary Watson-Crick base pairing, any off-target interactions can be predicted bioinformatically, and adverse effects that these interactions may cause can be forecasted and potentially circumvented.

An increased understanding of the roles of genes and other oligonucleotides in disease is leading to an expansion of opportunities for developing oligonucleotide therapies directed to previously undruggable targets. A particular focus of oligonucleotide therapy is oncology, with approximately one-quarter of oligonucleotide therapy candidates being developed to modulate targets in cancer.^56^ Given that TSGs are commonly downregulated in cancer, and small molecules have traditionally been used to inhibit, rather than stimulate, a target, there is the potential to restore TSG expression and function using stimulatory oligonucleotides, such as saRNAs and synthetic nucleoside-modified mRNAs. The remainder of this review describes these forms of oligonucleotide therapy and summarizes the current evidence that they can be used to modulate TSG expression in the context of cancer.

## Different approaches to restoring tumour suppressor function

### saRNAs

saRNAs are an emerging sub-class of oligonucleotides that have the potential to stimulate the transcription, and hence increase the expression, of a target gene. Unlike siRNAs, which work by interacting with mRNA to block its translation or mediate its degradation, saRNAs instead mediate transcriptional activation by binding to the complementary sequence in or near the promoter region of the target gene.^57^ saRNAs are short, 21-nucleotide, double-stranded oligomers that are typically delivered to the target organ encapsulated in a lipid nanoparticle. There are many research groups interested in using saRNAs to upregulate TSGs which, as discussed above, are commonly downregulated in various forms of cancer. To date, there is a single saRNA candidate that has progressed into clinical trials, targeting the *CEBPA* gene in hepatocellular carcinoma (HCC). However, there are many other TSGs being explored as potential saRNA targets preclinically (Table 1).

### Mechanism of action

Once an saRNA has entered the cell, the guide strand of the double-stranded molecule is loaded into argonaut protein 2 (Ago2).^2^ The guide strand of the saRNA duplex is determined by the thermodynamic stability of the 5′ end of the molecule and is usually the antisense strand of the complex.^58^^,^^59^^,^^60^ Chu et al.^58^ (2010) showed that siRNA repression of Ago2 expression significantly decreased saRNA activity, but did not diminish it completely, suggesting there may be a secondary, currently unknown mechanism that also facilitates saRNA delivery to the nucleus.

**Figure 2:**
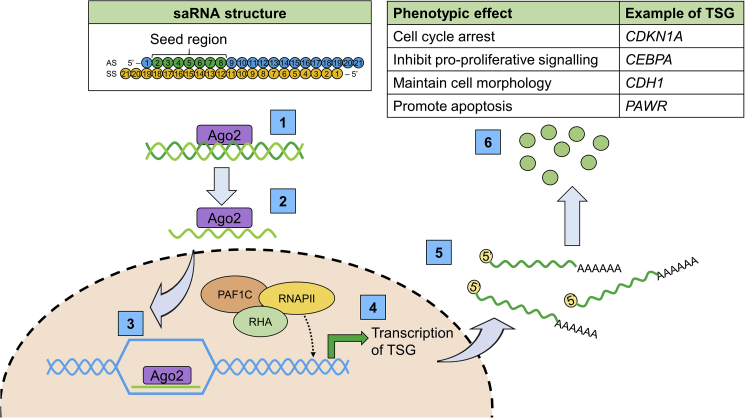
saRNA structure and mechanism saRNAs are small, 21 oligonucleotide double-stranded molecules. Once the saRNA has entered the target cell, the double-strand molecule is loaded onto Ago2 **(1)**, and the passenger strand (usually the sense strand of the complex) is released and degraded **(2)**. The Ago2/guide strand complex translocates into the nucleus, facilitated by transporters such as importin-8 and the guide strand interacts with the complementary sequence within the promoter region of the target gene **(3)**. This interaction facilitates recruitment of transcriptional machinery including RHA, PAF1C, and RNA polymerase II (RNAPII) to stimulate transcription of the target gene **(4)**, resulting in an increase in target gene mRNA **(5)** and functional protein **(6)**.

saRNA-loaded Ago2 protein is shuttled to the nucleus via importin-8,^61^ and the saRNA guide strand interacts with the complementary sequence in the promoter region of the target gene. This interaction facilitates the recruitment of other proteins required for transcriptional activation, including proteins involved in RNA splicing and binding such as heterogeneous nuclear ribonucleoproteins.^60^ Others include RNA helicase A (RHA) and RNA polymerase II complex component 9 (CTR9), both of which are known transcriptional activators. RHA is a helicase that catalyzes the unwinding of double-stranded DNA and acts as a bridging factor linking β-actin and RNA polymerase II to form part of the pre-initiation complex.^62^ CTR9 forms part of the polymerase-associated factor 1 complex (PAF1C), which can interact with histone-modifying enzymes and RNA polymerase II directly to activate gene transcription.^60^ PAF1C can induce mono-ubiquitination of histone 2B and recruit methyltransferase enzymes to methylate histone 3 lysine 4 (H3K4). H3K4 methylation results in the DNA becoming less condensed around the histone nucleosome, thus making the promoter more accessible for the transcriptional machinery and leading to an increase in gene expression (Figure 2).^3^

### Targeting TSGs using saRNAs

saRNAs were discovered by two groups in tandem. Janowski et al.^63^ (2007) discovered them during the development of anti-gene oligonucleotides (agRNAs) designed to downregulate the expression of the progesterone receptor (PR) in breast cancer. Unexpectedly, the group found that some of their synthesized oligonucleotides actually increased the expression of PR by up to 2-fold in T47D and MCF7 cells. The authors also showed that selected agRNAs targeting major vault protein could upregulate its protein expression by up to 4-fold.^63^ At a similar time, Li et al.^2^ (2006) successfully developed saRNAs targeting *CDH1*, *CDKN1A*, and *VEGF* in prostate cancer cell lines. Since their initial discovery, there has been a number of groups interested in using saRNAs to target and upregulate genes in specific diseases, in particular, targeting TSGs in cancer. TSGs that function in different capacities to avert cancer, such as by inducing cell-cycle arrest, preventing pro-proliferative signaling, maintaining cell morphology, and inducing apoptosis have been targeted using saRNAs, and examples from each classification are discussed below. Further examples can be found in Table 1.

### Cell-cycle arrest: *CDKN1A*

A TSG that is commonly downregulated in cancer is cyclin-dependent kinase inhibitor 1A (*CDKN1A*), which encodes p21. p21 is involved in controlling the cell cycle and is able to induce cell-cycle arrest by preventing cyclin-dependent kinase 4 and cyclin D1 from forming an active kinase complex. This prevents the RB protein from becoming phosphorylated and, thus, prevents transcription factor E2F from being active and driving the transcription of genes involved in the S-phase of the cell cycle, such as DNA polymerase and cyclin A.^4^ p21 can also induce apoptosis by suppressing the pro-apoptotic protein B-cell lymphoma-extra-large and activating caspase-3 and poly-ADP ribose polymerase.^4^ Therefore, because of the anti-proliferative and pro-apoptotic roles of p21, there has been interest in targeting this protein in oncology.

Since the initial discovery of *CDKN1A* saRNAs by Li et al.^2^ (2006), Place et al.^5^ (2012) showed that addition of 2′ fluoro modifications to the pyrimidine bases in the guide strand increases the stability of the saRNA while retaining RNA activation activity. The saRNA developed by Place et al.^5^ (2012) also successfully upregulated p21 in a mouse prostate cancer tumor model leading to significant anti-tumour activity. Another study has tested the efficacy of p21 saRNA *in vitro* in many different cancer cell lines, including PC3 (prostate), T24 (bladder), ACHN (renal), U2-OS (osteosarcoma), and 293T (embryonic kidney).^3^ The saRNA was able to upregulate transcriptional activity at the p21 promoter by increasing methylation at H3K4.^3^

A number of saRNAs targeting *CDKN1A* have been developed and shown to successfully upregulate gene expression at both the mRNA and protein level. Furthermore, the upregulation of p21 leads to changes in cell behavior; in colorectal cancer, HCC, prostate cancer, and renal cell carcinoma cell lines, p21 upregulation results in more cells residing in G0 or G1 phase of the cell cycle,^6^^,^^7^^,^^8^^,^^64^ as well as a decrease in migrative and invasive capabilities in prostate cancer cell lines.^8^

### Inhibition of pro-proliferative signaling: *CEBPA*

The first saRNA candidate to enter human clinical trials targets the *CEBPA* gene, which encodes CCAAT/enhancer-binding protein alpha (C/EBP-α), one of six members of the C/EBP transcription factor family. C/EBP-α upregulates expression of albumin (*ALB*), adipose triglyceride lipase (*ATGL*), colony stimulating factor 3 receptor (*CSF3R*), *CDKN1A*, and glycogen synthase 1 (*GYS1*), among other genes.^57^ These targets encode proteins that are involved in preventing the over-proliferation of mature hepatocytes and maintaining healthy hepatocyte metabolic function. C/EBP-α also downregulates expression of pro-proliferative and pro-inflammatory genes such as *c-MYC* and interferon-gamma (*INFG*).^57^ C/EBP-α expression is decreased in 60% of HCC tumors in comparison with non-tumour tissue from the same patients, and its deficiency is associated with increased hepatic proliferation in mice, thus resulting in worsened tumor phenotype.^65^

*In vitro* studies showed that increased *CEBPA* expression in HCC cell lines led to decreased expression of mesenchymal markers such as N-cadherin, Slug, and vimentin and upregulated epithelial markers such as E-cadherin, implying that the upregulation of C/EBP-α helps to prevent epithelial-to-mesenchymal transition.^14^ The upregulation of C/EBP-α also resulted in the downregulation of other pro-proliferative proteins such as β-catenin and epidermal growth factor receptor (*EGFR*), *c-MYC*, axis inhibition protein 2 (*AXIN2*), and cyclin D1 (*CCND1*).^14^

Huan et al.^14^ (2016) investigated the effects of saRNA-targeting *CEBPA* in a mouse orthotopic HCC tumor model. Over 40 days, the group saw a decrease in the tumor size and intrahepatic metastasis in CEBPA saRNA-treated mice compared with mice treated with a scrambled saRNA. Furthermore, the downregulation of pro-proliferative genes *EGFR* and catenin beta 1 (*CTNNB1*) was observed in CEBPA-saRNA-treated mice using immunohistochemistry analysis.^14^

In another study, CEBPA-51, the preclinical candidate saRNA targeting *CEBPA*, was encapsulated into liposomal nanoparticles (SMARTICLES) for targeted delivery to the liver, and injected into rats exhibiting cirrhotic HCC, induced by injection of diethylnitrosamine over a 9-week period.^66^ Animals treated with saRNA targeting CEBPA had an 80% decrease in hepatic tumor size compared with animals treated with a non-specific oligonucleotide.

Recently, MTL-CEBPA completed first-in-human clinical trials to assess tolerability in patients. The trial consisted of 34 patients with advanced HCC with underlying cirrhosis, metastasis or resulting from non-alcoholic steatohepatitis. Despite the drug not causing a significant improvement in liver function test results compared with baseline after two cycles of treatment, the relative expression of CEBPA mRNA in white blood cells increased 1.5-fold consistently across all treatment groups.^15^ Furthermore, one patient achieved a confirmed partial tumor response, as seen both by computed tomography scan and by a rapid decrease in the alpha-fetoprotein level, which was maintained for 24 months. The mean progression-free survival of the entire patient cohort (different doses of MTL-CEBPA) was 4.6 months, suggesting that the drug has some anti-tumour activity.^15^

A phase Ib clinical trial of MTL-CEBPA in combination with sorafenib in patients with advanced HCC showed that the treatment can successfully decrease the tumor burden. One-quarter of patients who were naive to tyrosine-kinase inhibitor treatment and had a viral etiology showed a complete or partial objective response to treatment after 12 months, with three patients exhibiting a complete response, as demonstrated by a complete eradication of target lesions at month 12.^16^ Furthermore, the study demonstrated that the upregulation of *CEBPA* expression caused by the treatment leads to the downregulation of immune suppressive genes in myeloid-derived suppressor cells and the upregulation of genes associated with monocyte and neutrophil function.^16^ Although no upregulation in monocyte and neutrophil levels was seen in the patients’ blood 24 h and 7 days after MTL-CEBPA administration, a marked decrease in the levels of monocytic MDSCs was observed. This suggests that MTL-CEBPA is able to exert its tumor suppressive effects by abrogating the immune suppressive activity of monocytic MDSCs in the tumor microenvironment.^16^

Given the success of MTL-CEBPA in clinical trials for advanced HCC, the drug has attracted interest as a novel therapeutic agent in other cancers, such as pancreatic ductal adenocarcinoma, for which saRNA targeting CEBPA has been reported to have anti-tumour effects both *in vitro* and *in vivo.*^17^^,^^18^

### Maintaining cell morphology: *CDH1*

*CDH1* is a TSG that has gained attention for its potential to be upregulated using saRNAs and has been investigated in this context across many cancer types.^2^^,^^23^^,^^24^^,^^25^^,^^26^^,^^27^ *CDH1* encodes the transmembrane glycoprotein E-cadherin, which is involved in maintaining epithelial cell morphology via adherens junction connections.^67^ Furthermore, E-cadherin is anchored to the cell’s cytoskeleton via an interaction with β-catenin, a key protein involved in the pro-proliferative Wnt signaling pathway.^68^^,^^69^ saRNAs targeting *CDH1* were initially discovered in 2006 in the context of prostate cancer.^2^ Wu et al. (2016) further investigated the effects of saRNAs targeting *CDH1* in prostate cancer, using different saRNAs to those designed by Li et al. (2006). The group found that one of their candidates, an saRNA that binds to the −661 region of the *CDH1* promoter, could successfully upregulate *CDH1* expression by up to 5-fold at the mRNA level, and that the anti-sense strand of the saRNA duplex was responsible for the RNA activating activity. The same group tested this saRNA in HCC cell lines and observed a similar increase in E-cadherin protein, as observed by western blot.

The −215 region of the *CDH1* promoter has also been commonly targeted, with saRNAs designed around this region being shown to successfully upregulate *CDH1* in renal carcinoma and breast cancer, and produce beneficial downstream phenotypic effects.^23^^,^^26^ Dai et al.^23^ (2018) found that renal carcinoma cell lines transfected with *CDH1* saRNA had a reduced migration capability compared with non-transfected cells, as measured using a Matrigel invasion chamber assay. When *CDH1* was knocked down again using RNA interference, the renal carcinoma cells regained their migration capability.^23^ Similarly, saRNAs targeting the −215 region of the *CDH1* promoter in breast cancer led to cells having a decreased proliferation rate, increased apoptosis and decreased migration capabilities, compared with mock-transfected and control sequence transfected cells, as well as a potent growth inhibitory effect *in vivo*.^26^

### Promoting apoptosis: *PAWR*

An example of a TSG which is involved in promoting apoptosis is PRKC apoptosis WT1 regulator (*PAWR*). This TSG has been implicated in the apoptotic response of prostate cancer cells to several exogenous agents. One group has investigated the impact of saRNAs targeting *PAWR in vitro* and has found that transfection with the oligonucleotide results in a decrease in cell growth, as well as cell shrinkage and apoptosis.^31^ This study provides proof-of-concept that *PAWR* upregulation in prostate cancer is a potential therapeutic avenue to explore, and provides opportunities for further *in vivo* work to build upon the preclinical profile of such a molecule.

## Synthetic mRNAs

Although saRNAs are a promising novel approach for upregulating TSGs in cancer, their ability to increase gene expression is limited to the upregulation of genes in which at least one allele retains normal activity. When both alleles of a gene are deleted or mutated, saRNAs are unable to exert any beneficial effect. An alternative oligonucleotide therapy that can be used to restore the expression of TSGs is synthetic, nucleoside-modified mRNA. Synthetic mRNAs have been explored for many years for their potential in cancer immunotherapy and have recently made headlines for their use in the development of sever acute respiratory syndrome coronavirus 2 vaccines.^70^^,^^71^^,^^72^ Synthetic mRNAs are synthesized to mimic natural mRNAs and are translated in the cytoplasm to produce protein, which is indistinguishable to that resulting from the translation of endogenous mRNA.

The *in vitro* mRNA synthesis process involves ligating the open reading frame of the gene of interest, along with any other desired sequences such as 5′ and 3′ untranslated regions into a plasmid with a viral promoter, usually T7, upstream of the transcription start site. The plasmid must be linearized, such that RNA transcripts of defined length are produced. The viral promoter is recognized by phage RNA polymerases to initiate RNA synthesis. These sequences are then transcribed *in vitro* to produce mRNA.^73^ The use of synthetic mRNAs to increase translation of a target protein has been under investigation since the 1980s. Malone et al.^73^ (1989) successfully transfected *in vitro* transcribed mRNA encoding *Photinus pyralis* luciferase into NIH 3T3 mouse cells. The same group showed that surrounding the open reading frame (ORF) of the target gene with 5′ and 3′ untranslated regions (UTRs) helped to increase translational efficiency.^73^

Given their size in comparison with saRNAs, synthetic mRNAs are more difficult to deliver to a cell; however, once inside the cell they can exert their effects rapidly, as the transcription stage is bypassed. However, the rapid degradation of mRNAs and the ability to activate toll-like receptors (TLRs) and stimulate the immune system led to a period of decline in interest in their use as a therapeutic agent.^74^

However, interest in the use of synthetic mRNAs was reignited in the mid-2000s, when Karikó et al. (2005)^75^ discovered that the addition of naturally modified nucleosides, such as pseudouridine and 5-methylcytidine, to the ORF resulted in a decreased secretion of cytokines and interleukin-8 in response to their administration *in vitro*. The modified mRNAs led to less stimulation of immune response mediators such as interferon-gamma, interleukin-2 (IL-12), interferon-alpha, retinoic acid-inducible gene 1, and TLRs 3, 7, and 8.^76^

**Figure 3:**
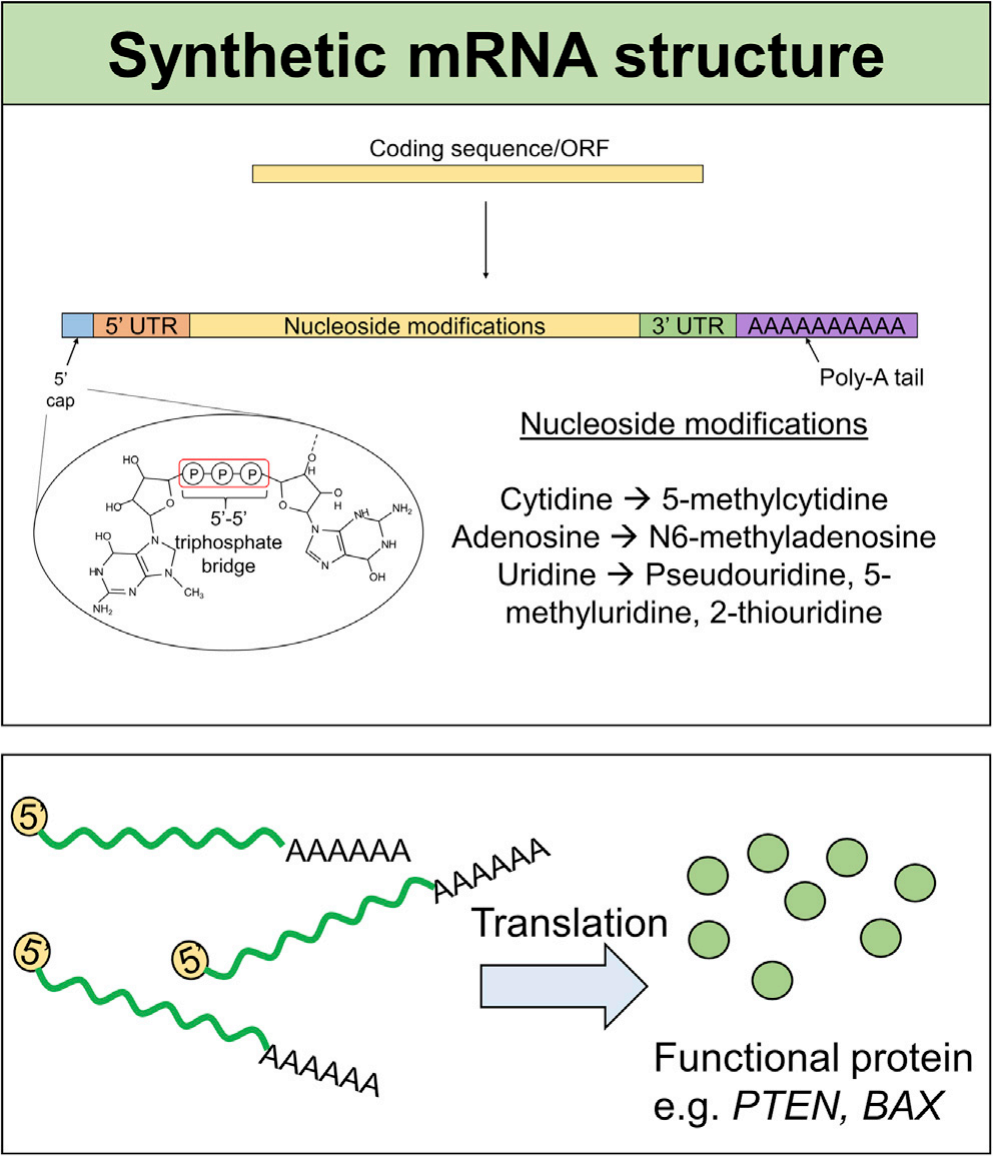
Synthetic mRNA structure and examples of their use in TSG upregulation Synthetic mRNAs are designed to mimic endogenous mRNAs by the addition of a 5′ cap, which contains a 5′-5′ triphosphate bridge to improve stability, 5′ and 3′ UTRs and a poly-A tail. The ORF of the structure can be further modified to include naturally modified bases such as 5-methylcytidine and N6-methyladenosine, which decrease immunogenicity and facilitate *in vivo* delivery. Examples of the use of synthetic mRNAs to restore TSG expression include *PTEN* in prostate cancer and melanoma and *BAX* in malignant melanoma.

Alongside the addition of 5′ and 3′ UTRs and nucleoside modifications to the ORF, synthetic mRNAs can be further modified with the addition of a 5′ cap and poly-A tail to mimic endogenous mRNAs (Figure 3). Endogenous mRNAs contain a single 7-methylguanosine residue at their 5′ end, attached to the next residue by a 5′-5′ triphosphate linkage.^77^ The 5′ cap confers resistance to enzymatic cleavage and allows for the translational machinery to recognise and bind to the mRNAs to allow efficient translation into protein.^73^

The 3′ poly(A) tail plays a role in improving mRNA stability. Optimization studies have shown that an increased length of poly(A) tail from 20 nucleotides to 120 nucleotides is associated with improved translational efficiency and therefore protein expression.^78^ However, other studies have used even longer poly(A) tails (e.g., ≤200 nucleotides in length) with similar success.^79^

### Using synthetic mRNAs to upregulate TSGs in cancer

Given the recent improvements in the stability and immunogenicity of synthetic mRNAs and the emergence of delivery systems such as lipid nanoparticles to facilitate the entry of large synthetic mRNAs into the cell, research into the use of such nucleoside-modified, *in vitro* transcribed mRNAs as therapeutic agents has become more established. mRNA therapy provides an advantage over small molecule drugs because of its ability to increase or restore protein expression, and therefore activity, whereas small molecules target proteins that are already present, often in an inhibitory manner. A particular area that has gained much traction in recent years is cancer immunotherapy.^80^^,^^81^^,^^82^ Alongside this, some groups have started to investigate the potential of using synthetic mRNAs to upregulate or restore the function of the TSGs that are commonly downregulated or lost in various cancers. Although synthetic mRNAs are a promising area of research and restoring gene function in other disease contexts has been explored extensively, such as *CFTR* in cystic fibrosis,^83^ research into the use of synthetic mRNAs to restore TSGs is currently somewhat lacking. This review discusses the literature demonstrating the potential of using synthetic mRNAs as a means of restoring TSG function in the context of cancer.

#### PTEN in prostate cancer and melanoma

Islam et al.^84^ (2018) have delivered synthetic mRNA encoding *PTEN* encapsulated in hybrid lipid nanoparticles into the prostate cancer cell line PC3. The hybrid nanoparticles were prepared using cationic lipid-like compound G0-C14 (for mRNA complexation) and poly(lactic-*co*-glycolic acid) polymer coated with a lipid-polyethyl glycol (PEG) shell to make a stable nanoparticle core.^84^ The group showed that transfection of the encapsulated mRNA was more effective than transfection of naked mRNA using a commercial cationic lipid transfection reagent. An increase in PTEN protein expression was seen 48 h after transfection, alongside a decrease in cell viability. Furthermore, the synthetic *PTEN* mRNA provoked a downregulation of the PI3K-AKT signaling pathway, as demonstrated by decreased phosphorylation of eukaryotic translation initiation factor 4E-binding protein 1, proline-rich AKT substrate of 40 kDa, and Foxo3a.^84^ Decreased phosphorylation of these proteins indicates the inhibition of AKT activity and its downstream pro-proliferative and pro-survival effects. *In vivo* injection of the *PTEN* mRNA encapsulated in nanoparticles caused a suppression of growth of PC3 xenograft tumors compared with control injections of PBS or mRNA encoding *EGFR*.^84^

The same group went on to develop a nanoparticle containing *PTEN* mRNA for the delivery of the oligonucleotide into *Pten*-null or mutated tumor cells and explored the effects of such therapy on the tumor microenvironment.^85^ The treatment of *Pten*-deficient murine cells with nanoparticles containing *PTEN* mRNA led to the induction of apoptosis and autophagy pathways *in vitro*, as well as calreticulin exposure on the cell membrane and ATP release into the extracellular environment, indicating that *PTEN* upregulation may influence the tumor microenvironment. The hypothesis was explored further *in vivo* in a melanoma tumor model, and the group saw that injection of *PTEN* mRNA-containing nanoparticles led to an increase in CD3^+^CD8^+^ T cells and decrease in regulatory T cells and myeloid-derived suppressive cells, which helps to reverse the immunosuppressive tumor microenvironment.^85^ Furthermore, the upregulation of *PTEN* in melanoma and prostate cancer tumor models led to an increase in efficacy of anti-programmed cell death protein 1 therapy, highlighting the potential benefits that activating oligonucleotides can have in the clinical oncology field.^85^

#### Bax in malignant melanoma

Okumura et al. (2008) used cationic liposomes to deliver mRNA encoding Bax, a pro-apoptotic protein into HMG malignant melanoma cells. Bax stimulates the release of cytochrome C from the mitochondria, which in turn leads to the initiation of apoptosis.^86^^,^^87^ The group found that encapsulation of the mRNA into cationic liposomes was sufficient to complex the mRNA and deliver it to the cells. Furthermore, an increase in the levels of Bax protein and caspase-3 activity were observed 24 h after transfection, alongside a significant decrease in cell survival.^88^ The only modifications added to the synthetic mRNA were anti-reverse cap analogs (ARCA) 5′ cap and poly(A) tail. ARCAs are commonly used in the synthesis of commercial mRNAs to increase translational efficiency.^89^ They prevent incorporation of the 5′ cap in the reverse orientation, which would stop the mRNA from being recognized by the ribosome and translated.

## Benefits and challenges of using different oligonucleotide therapies

saRNAs and synthetic mRNAs are two types of oligonucleotide therapy that can be used to upregulate the expression of TSGs in cancer. However, they differ in terms of size and mechanism of action, as well as their potential for gene upregulation (Table 2). Given that saRNA activity requires entry into the nucleus and interaction with the promoter region of the target gene, saRNAs have the potential to upregulate only functional genes. In contrast, synthetic mRNAs operate within the cytoplasm and provide the cell with the correct mRNA sequence, which is then directly translated into functional protein. This allows for synthetic mRNAs to be used to restore otherwise absent proteins in disease, as evidenced by the use of this approach in protein replacement therapies, such as mRNA encoding the cystic fibrosis transmembrane conductance regulator protein in cystic fibrosis.^83^ The requirement for increased transcription of endogenous DNA in the saRNA mechanism means that protein production is slow, taking up to 72 h in many *in vitro* models.^19^ However, protein production can be seen within 24 h of administration of synthetic mRNAs.^90^^,^^91^^,^^92^

**Table 2:** Comparison of saRNAs and synthetic mRNAs as agents for modulation of tumor suppressor activity in cancer

	saRNAs	Synthetic mRNAs
Size	Small, 21 oligonucleotides	Long, 1,000s of oligonucleotides
Active site	Nucleus	Cytoplasm
Delivery	*In vitro* – standard transfection reagents *In vivo* – lipid nanoparticles	*In vitro* and *in vivo* – lipid nanoparticles
Onset of effect	Slower (within 72 h of administration) due to requirement for transcription Li et al., Reebye et al. (Refs.^2^^,^^19^)	Faster (within 24 h of administration) due to rapid translation Pardi et al., Rizvi et al. (Refs.^90^^,^^91^^,^^92^)
Duration of effect	Weeks to months with stabilized saRNA, requiring infrequent dosing for therapeutic effects Hashimoto et al. (Ref.^16^)	Days, requiring frequent dosing for prolonged therapeutic effects (except vaccines).
Safety	mRNA upregulated within normal range effect restricted to cells that normally express target gene	Risk of overexpression. Effect not restricted to cells that normally express target gene. Potential mitochondrial toxicities (due to addition of unnatural nucleoside analogues). Sahin et al. (Ref.^93^)

A major hurdle in translating the use of these classes of oligonucleotide therapy into the clinic is the delivery of the oligonucleotides *in vivo*. Some of the challenges faced by oligonucleotides include degradation by nucleases in the extracellular space, renal clearance, crossing the capillary endothelium once at the target organ, crossing the cell membrane, and lysosomal degradation.^94^ Oligonucleotides are hydrophilic and so are unable to penetrate the plasma membrane without assistance. Furthermore, the introduction of any foreign oligonucleotide can result in stimulation of the innate immune system and TLRs. Therefore, a suitable delivery system must be used to ensure that the oligonucleotide reaches its target site, and enters the target cell before it is degraded by extracellular nucleases or stimulates the innate immune response.

The most common non-viral delivery system used for oligonucleotide therapies is lipid nanoparticles. This process involves complexing the anionic oligonucleotide with cationic lipids to produce a nanoparticle. For *in vivo* delivery, more complex structures may be used to prevent the nanoparticle from being cleared from the circulation by reticuloendothelial system phagocytes. For example, the surface can be coated with a neutral polymer such as PEG to prevent protein adherence to the nanoparticle and clearance.^95^ saRNAs are small structures and can be delivered *in vitro* using simple transfection reagents. However, synthetic mRNAs are much larger and require encapsulation within a nanoparticle for both *in vitro* and *in vivo* delivery (Table 2).

Another method for oligonucleotide delivery that can be used as an alternative to nanoparticles, or in conjunction with them, is bioconjugation. This is where the oligonucleotide or carrier (i.e., nanoparticle) is conjugated to a ligand that promotes interaction of the molecule with the target cell. For example, a commonly used ligand for selective delivery of oligonucleotides to the liver is N-acetylgalactosamine (GalNAc). The GalNAc ligand is recognized by the asiaglycoprotein receptor, which is highly expressed on the surface of hepatocytes and internalizes the ligand via Ca^2+^-dependent endocytosis.^96^^,^^97^ The GalNAc ligand can be directly attached to small oligonucleotides, such as saRNAs and siRNAs, but this is not possible for larger molecules such as synthetic mRNAs, which require encapsulation within a nanoparticle for both *in vitro* and *in vivo* delivery. Some oligonucleotide therapies approved by the U.S. Food and Drug Administration use the GalNAc conjugation system, such as givosiran, an siRNA to treat acute hepatic porphyria.^98^

## Conclusions and future perspectives

TSGs are commonly downregulated in cancer and, despite the idea gaining much attention within the scientific community, efforts to upregulate or reactivate TSGs thus far have remained relatively fruitless. However, emerging classes of therapy, such as oligonucleotide therapies, offer an advantage over traditional small molecule drugs in their capability to work at the gene level, rather than by targeting a protein. Oligonucleotide therapies have the potential to be combined with small molecules or other oligonucleotides to produce a synergistic effect. saRNAs and nucleoside-modified RNAs are two classes of oligonucleotide therapy with the potential to upregulate expression of target genes, or in the case of synthetic mRNAs, replace a lost or faulty protein in cancer. saRNAs offer a benefit over synthetic mRNAs in terms of their size and ease of delivery; however, they are an inappropriate treatment option for patients with a mutated allele of the target, as the upregulation of a mutated target will not have a beneficial effect and may even be detrimental. Future work should aim to address challenges relating to delivery and immunogenicity to improve the potential of oligonucleotide therapies to progress through clinical trials and to meet the need for TSG upregulation in cancer. Furthermore, as targeted oligonucleotide therapies start to emerge into the clinic, the use of tumor genome sequencing could help to match a patient with an ideal therapy based on the genetic profile of their tumor. The field of research is still in its infancy and provides exciting opportunities for development and clinical translation in the coming years.
